# Position statement on the management of pregnancy in sickle cell disease

**DOI:** 10.1111/ajo.13888

**Published:** 2024-09-27

**Authors:** Mimi Yue, Kylie Mason, Shelley Rowlands, Zane Kaplan, Debra Kennedy, Giselle Kidson‐Gerber

**Affiliations:** ^1^ Department of Haematology Mater Health Service Brisbane Queensland Australia; ^2^ School of Medicine University of Queensland Brisbane Queensland Australia; ^3^ Department of Clinical Haematology Peter MacCallum Cancer Centre and the Royal Melbourne Hospital Melbourne Victoria Australia; ^4^ Sir Peter MacCallum Department of Oncology University of Melbourne Melbourne Victoria Australia; ^5^ Department of Maternal Fetal Medicine Royal Women's Hospital Melbourne Victoria Australia; ^6^ Monash Haematology Monash Health Melbourne Victoria Australia; ^7^ Department of Medicine, School of Clinical Sciences at Monash Health Monash University Melbourne Victoria Australia; ^8^ Royal Hospital for Women, Randwick Campus Sydney New South Wales Australia; ^9^ Schools of Medicine and Women's and Children's Health, Prince of Wales Hospital and Royal Hospital for Women Clinical School University of NSW Sydney New South Wales Australia; ^10^ Department of Haematology NSW Health Pathology, Prince of Wales Hospital and Royal Hospital for Women, Randwick Campus Sydney New South Wales Australia

**Keywords:** anaemia, pregnancy, pregnancy complications, prenatal care, sickle cell

## Abstract

Sickle cell disease (SCD) is a hereditary haemoglobinopathy which causes multi‐organ dysfunction. Pregnancies in SCD are high risk with significant maternal and fetal morbidity and mortality, including vaso‐occlusive crises, thrombosis, anaemia, placental insufficiency, fetal growth restriction, preterm birth and medication effects. High level evidence on this topic is lacking. The Australian Sickle Cell Disease Working Group has reviewed international guidelines on this topic and provide an up‐to‐date and structured approach to the pre‐conception, antenatal, birth and post‐partum management of these women. Early and comprehensive multidisciplinary care involving experienced clinicians is recommended.

## INTRODUCTION

Sickle cell disease (SCD) is an autosomal recessive, inherited disorder of haemoglobin (Hb). While still considered a relatively rare disease in Australia, prevalence is increasing as are pregnancies in women with SCD. Pregnancy in women with SCD is associated with a significantly increased risk of maternal and fetal mortality and morbidity.

It is estimated that approximately 300 000 babies are born each year with SCD worldwide, with the majority being in low‐ to middle‐income countries.^1^ Currently 359 persons with a sickling disorder are recorded in the Australian Haemoglobinopathy Registry. However, it is likely the data are an underestimation due to under‐reporting and incomplete participation in the registry.^2^ The prevalence of SCD in Australia is predicted to increase due to migration and improved life expectancy in high‐income countries.^3^ Therefore, it is expected there will be increasing numbers of pregnant women with SCD seeking medical care in Australia.

Sickle haemoglobin (HbS) results from a point mutation in the beta globin gene, which leads to a single amino acid substitution (Glu6Val) in the beta globin subunit. This amino acid change results in the propensity for HbS to polymerise in the deoxygenated state and consequent sickling of red blood cells.^4^ The sickled red cells are inflexible and lead to red cell haemolysis, vaso‐occlusion, inflammation, hypercoagulability through endothelium activation, ischaemia and subsequent end organ damage. Acute vaso‐occlusive events lead to episodes of acute pain and tissue damage (pain crisis). Repeated vaso‐occlusive crises (VOC) lead to permanent damage in all organ systems. eg stroke, chronic kidney disease, pulmonary hypertension and avascular necrosis.^5^

**Figure 1:**
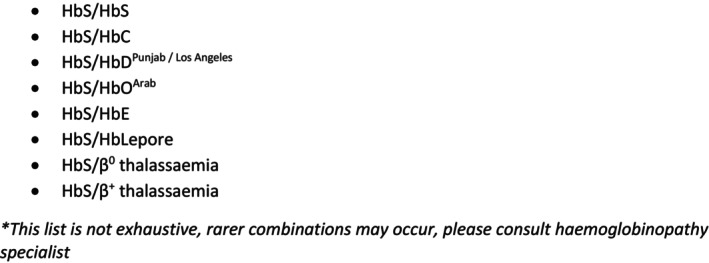
Significant haemoglobinopathy genotypes associated with sickle cell disease (adapted from (7)).

While sickle cell anaemia refers to homozogosity for HbS, SCD may arise from either HbS homozygosity or compound heterozygosity for HbS together with either a beta thalassaemia mutation or another beta globin variant. The spectrum of implicated partner haemoglobinopathies include beta thalassaemia (Hbß^0/+^), HbE, HbO^Arab^, HbD, Hb Lepore and HbC (Fig. 1). Correct molecular characterisation of the partner gene is imperative as it has significant phenotypic implications.

Pregnancy in women with SCD is associated with an increased risk of both maternal and fetal morbidity (Table 1). The physiological changes of pregnancy (prothrombotic state, increased metabolic demand and reduced immunity) increase vulnerability to complications. Women with pre‐existing medical problems, including those from SCD, are at additional risk. VOC disease may result in placental disorders including placental insufficiency with increased risk of miscarriage, abruption, pre‐eclampsia, eclampsia, fetal growth restriction, preterm birth, fetal death *in utero*.^6^, ^7^

**Table 1:** Potential complications in sickle cell disease (SCD) during pregnancy

	Risks
Effect of SCD on maternal health prior to pregnancy	End organ damage, eg cardiomyopathy, chronic kidney disease, pulmonary hypertension, avascular necrosis of hip Iron overload Chronic pain
Effect of SCD on pregnant mother	Increased risk of vaso‐occlusive crises Increased risk of thrombosis Worsening anaemia Increased risk of infection Increased risk of placental insufficiency‐related problems – miscarriage, abruption, pre‐eclampsia, fetal effects (see below)
Effect of SCD on fetus	Fetal growth restriction Preterm birth Fetal death *in utero*
Effect of medications on fetus	Refer to Table 2

This position statement provides guidelines for current best practice in the management of SCD during pregnancy in the Australian context, as optimisation of management in pregnancy will result in better outcomes for mother and baby. The primary audience is haematologists, obstetricians, maternal‐fetal medicine specialists, clinical nurse consultants, clinical nurse educators and associated allied health practitioners, with additional clinicians, such as general practitioners, emergency physicians, general physicians, associated subspecialists and rural practitioners who may provide care in collaborations with the primary treating team. This position statement has been endorsed by the Royal Australian and New Zealand College of Obstetricians and Gynaecologists (RANZCOG), Society of Obstetric Medicine of Australia and New Zealand (SOMANZ), Haematology in Obstetrics and Women's Health Collaborative (HOW Collaborative), and the Haematology Society of Australia and New Zealand (HSANZ).

## MATERIALS AND METHODS

International consensus guidelines and expert position statements^7^, ^8^, ^9^, ^10^, ^11^, ^12^ were reviewed. Application to practice in the Australian healthcare context was considered, with reference to local guidelines and standards. The recommendations were discussed and endorsed by a national panel of expert SCD clinicians.

## AUSTRALIAN CONTEXT/APPLICATION

Australia is a high‐income country with good access to medications, advanced procedures such as automated red cell exchange transfusion and advanced obstetric medical services. The Australian healthcare funding structure is complex, with funding from national as well as state/territory governments, and non‐government sources such as private health insurers. Access to safe blood products is coordinated by a national organisation, Australian Red Cross Lifeblood. The blood donor pool is largely Caucasian, which makes it challenging to find phenotype‐matched blood in phenotypes more common in other ethnic groups, which are overrepresented in the SCD patient cohort.

SCD is considered a rare disease in the Australian community. Most health professionals, including obstetricians and haematologists, will have limited experience in managing SCD patients. Therefore, it is recommended that SCD pregnancies be managed in centres with experience in SCD and high‐risk pregnancies.

## RECOMMENDATIONS

### Pre‐conception

Pregnancy outcomes for mother and baby are improved when a woman is in optimal health going into pregnancy. A reproductive health plan should be reviewed bi‐annually with a pre‐conception consultation implemented to optimise the mother's health.

1. Screening and genetic testing.
  - Establish genotype/phenotype of the woman and partner prior to pregnancy to identify at‐risk combinations (Fig. 1), which may include full blood count, iron studies, haemoglobinopathy testing and molecular assessment of the alpha and beta globin genes.
  - If identified as an at‐risk couple, refer for genetic counselling and molecular testing. Discuss reproductive options, including non‐intervention, invasive prenatal diagnosis or *in vitro* fertilisation (IVF) and pre‐implantation genetic diagnosis. Because options involving molecular testing take time, early referral is recommended.
2. Counsel woman that there is an increased risk of complications for both mother and baby during pregnancy (Table 1).
3. Counsel woman that there is potentially a reduced fertility window due to accelerated age‐associated decline in ovarian reserve and consider early referral to reproductive services.

4 Pre‐conception comprehensive review overseen by a specialist multidisciplinary coordinated team (MDT) with experience managing these women (Table 2).
  - Optimise organ function.
  - Review medications and vaccination status.
    - *Note on hydroxyurea therapy*: Decisions to continue or cease hydroxyurea should be made in consultation with the MDT experienced in the management of SCD, balancing the benefits of treatment of SCD for mother and fetus with the theoretical risks of exposure of the fetus to hydroxyurea. There is limited but reassuring data about use of hydroxyurea in pregnancy, including during the period of organogenesis, with no significant signal of congenital malformation/teratogenicity.^13^, ^14^, ^15^ It is important to individualise counselling and advice based on individual maternal health needs and disease phenotype.
  - Pre‐transfusion work up – blood group genotype and antibody screen.
  - Folic acid 5 mg daily.
  - Review and update the reproductive health plan.
5 Consider referral to appropriate subspecialty for additional pre‐pregnancy counselling: pregnancy unit with experience in the management of SCD, fertility, maternal‐fetal medicine (MFM), pharmacy, cardiology, nephrology and endocrinology.

**Table 2:** Comprehensive review and sickle cell disease (SCD) specific management for pregnancy care (note: standard obstetric investigations should also be completed as part of routine care)

Review/assess	Action
End organ function	
Cardiac function Pulmonary hypertension	Echocardiography
Chronic lung disease	Pulse oximetry Pulmonary function test
Renal function	Blood pressure Serum creatinine Urinary protein creatinine ratio, albumin creatinine ratio
Avascular necrosis (AVN) (hips)	Review history of AVN, may affect mode of birth
Stroke and silent cerebral ischaemia	Transfusion program for primary or secondary prevention if history of stroke
Blood tests	
Iron status	Iron studies
Full blood count	Baseline Hb
Haemoglobinopathy tests	Baseline HbS, HbF
Blood group and antibody screen Red cell genotype (or phenotype if genotype not available)	Liaise with blood bank regarding transfusion plans during pregnancy and birth Partner red cell phenotype if maternal red cell antibodies
Red cell product requirements	ABO‐compatible RhD,C,c,E,e, Kell‐matched Antigen negative if allo‐immunised Consider extended matching if feasible Cytomegalovirus‐negative
Functional asplenia Encourage registration with Spleen Australia (spleen.org.au)	
Vaccines	Asplenic vaccinations (meningococcal, pneumococcal) Influenza vaccination Covid vaccination
Antibiotics	Prophylactic penicillin vs emergency supply/sick day plan
Medication review	
Acute/chronic pain medications	Update acute pain plan for pregnancy Paracetamol is safe in pregnancy Nonsteroidal anti‐inflammatories can be used between 12 and 20 weeks. Avoid after 20 weeks due to oligohydramnios risk^16^ Chronic pain team involvement for chronic opioid therapy
Hydroxyurea	If safe to discontinue during pregnancy, stop hydroxyurea when pregnancy confirmed. Consider commencement of red cell exchange or top‐up transfusion and consider restarting hydroxyurea after the first trimester. Continuing hydroxyurea therapy may be appropriate in some cases (see Pre‐conception Recommendation 3.b.i. for details).
Emerging therapies (not currently available in Australia)	Currently no safety data in pregnancy for the use of voxelotor, protein kinase inhibitors. Cease before attempting to conceive
Iron chelation	Stop when pregnancy confirmed
ACE inhibitors (ACEI) and angiotensin receptor blockers (ARB)	Switch to alternative antihypertensive prior to planned pregnancy. Stop ACEIs and ARBs when pregnancy confirmed and must be ceased by end of first trimester
Vitamins	Folic acid 5 mg daily Routine pregnancy multivitamin. If iron overload present, administer multivitamin without iron Vitamin D supplement if deficient
Aspirin	Pre‐eclampsia prophylaxis 150 mg daily when pregnancy confirmed to 36 weeks gestation
Venous thromboembolism (VTE) thromboprophylaxis	For example, enoxaparin 40 mg subcutaneous daily During all admissions unless contraindicated Antepartum – consider VTE thromboprophylaxis on a case‐by‐case basis based on risk profile Post‐partum VTE thromboprophylaxis is recommended for six weeks

### Management in pregnancy

#### General

1. Management by a specialist multidisciplinary coordinated team with experience managing pregnancy in SCD. Early referral recommended.
2. Assess and optimise as per Table 2 if not completed.
3. Confirm/establish haemoglobinopathy genotype of mother and partner within the first trimester, (see Pre‐conception Recommendations).
4. Monitoring as per Table 3.
5. Prompt treatment of crisis triggers – eg persistent vomiting and dehydration, associated with nausea and vomiting of pregnancy and hyperemesis gravidarum, infection.
6. Aspirin 150 mg daily from pregnancy confirmation to 36 weeks gestation.
7. Low threshold for admission.
8. Develop a written intra‐partum and post‐partum management plan in consultation with the mother, haematologist, MFM specialist, neonatologist and MDT team.

**Table 3:** Monitoring during pregnancy in sickle cell disease in addition to routine pregnancy care

Obstetric monitoring	Involve mother‐fetal medicine/high risk obstetric service Visits may be required more frequently Screen for Pre‐eclampsia Urinary infection (early pregnancy) Anaemia Be alert for dehydration infection Pain that is not obstetric
Haematology	Arrange regular visits throughout pregnancy
Other specialists, eg cardiologist, anaesthetist	As needed
Fetal monitoring	Dating scan in early pregnancy Screen for fetal growth restriction, eg monthly Serial growth scans from 24 weeks
Multidisciplinary agreement and documented plans	Plans for admissions, labour, birth and post‐partum care

#### Blood transfusions during pregnancy

1. Ensure blood group, antibody screen and red cell genotype (phenotype if genotype not available and patient has not been transfused in last three months) has been performed.
2. If transfusion is needed, pregnant women with SCD should be given ABO‐compatible, extended Rh (ie RhD, C, c, E, e) and Kell‐matched, cytomegalovirus‐negative units. Consider matching for Duffy as possible. If there are clinically significant red cell antibodies (current or historical) then the red cells selected should be negative for the corresponding antigens.

Pregnancy is not an indication for prophylactic blood transfusion. Women on transfusion therapy prior to conception should continue their routine therapy. Women who transition off hydroxyurea for or during pregnancy may require commencement of transfusion therapy to prevent SCD complications, including VOC. There is a preference for red cell exchange transfusion; however, top‐up transfusions are also acceptable.

#### Management of complications

##### Principles of management

1. Low threshold for admission.
2. Joint management by haematology, obstetrics, obstetric medicine – admitting team/ward determined by required skill set for stage of pregnancy and issue at hand.
3. Consider sickle‐related, obstetric and other non‐obstetric causes for presenting symptoms.

##### Management of acute VOC

1. Thorough assessment to determine if pain is due to VOC (eg typical pain crisis symptoms for that woman) or other cause in the obstetric setting.
2. Consult patient's agreed pain management plan if available.
3. Follow standard pain management guidelines and involvement of acute pain service.
4. Avoid nonsteroidal anti‐inflammatories in first and third trimesters, use judiciously between 12 and 20 weeks.
5. Low threshold for admission and venous thromboembolism (VTE) thromboprophylaxis.
6. Monitor fluid and oxygen balance carefully.
7. Have high level of vigilance for acute chest syndrome.

##### Management of VTE and thromboprophylaxis

1. VTE risk is increased throughout pregnancy and the post‐partum period.
2. Consider VTE thromboprophylaxis on a case‐by‐case basis.
3. Administer low molecular weight heparin for VTE thromboprophylaxis, as per usual guidelines.
4. Consideration of risks and benefits of anticoagulation, especially in the third trimester considering the risk of VTE vs risks such as placental abruption and unplanned early birth.
5. Management of VTE as per standard VTE guidelines in pregnancy.

### Pre‐delivery plan

- Ensure all members of MDT are aware of peripartum plan
- Anaesthetic review
- Anticoagulation plan
- Pain management
- Blood bank notification

### Intrapartum care (vaginal birth and caesarean section)

1. Late third trimester, intrapartum and post‐partum are periods of increased risk for complications.
2. Birth should occur in hospitals that are able to manage both the complications of SCD and high‐risk pregnancies in an MDT setting, with involvement of experienced clinicians.
3. Timing of birth should be directed by usual obstetric indications. There is no evidence to recommend a given gestation; however, birth by term should be considered due to the risk of late pregnancy complications.
4. Mode of birth should be directed by usual obstetric indications.
5. Consideration of positioning during labour/birth for women with hip/joint necrosis.
6. Communicate with blood bank to have extended matched red cell units available on admission.
7. Epidural is not contraindicated and may offer advantages during labour in SCD.
8. Avoid crisis precipitants in labour:
  - maintain hydration
  - keep warm
  - monitor vital signs and maintain oxygen saturations ≥97%
  - regular assessment of progress to avoid protracted labour
  - encourage incentive spirometry/deep breathing exercises.
9. Continuous fetal monitoring is directed by the usual indications.
10. Regional anaesthesia is preferred over general anaesthesia.
11. Consider an elective pre‐delivery red cell exchange in patients considered high risk of crisis.

### Post‐partum care, optimal post‐birth care

#### Mother

1. The postnatal period is a time of increased risk of pain. Precipitants should be anticipated and avoided:
  - maintain consistent analgesia, hydration and oxygenation, with early mobilisation
  - support adequate rest to minimise sleep deprivation.
2. Breastfeeding is not contraindicated. Use specialised services to assess exposures in breastfeeding, such as MotherSafe and LactMed.
3. Schedule recommencement of regular pre‐pregnancy medications.
4. VTE thromboprophylaxis for six weeks after birth.
5. Contraception should be individualised and discussed prior to discharge.

#### Baby

1. Screen for SCD in infant, if at risk.
2. Monitor for neonatal abstinence syndrome, haemolytic disease of the newborn if applicable.

## LIMITATIONS/OTHER CONSIDERATIONS

Due to limited literature available on this topic, this consensus statement is based on low‐level evidence and expert opinion. While these recommendations are considered best practice in the Australian setting, practice between centres may vary depending on availability of resources.

Reproductive issues in SCD, including subfertility in women and men are beyond the scope of this consensus statement and are comprehensively addressed elsewhere.^17^, ^18^

## CONCLUSION

Early and comprehensive multidisciplinary care involving experienced clinicians, nurses, midwifes and other allied healthcare professionals, is pivotal in achieving optimal outcomes for women with SCD and their infants in both the antenatal and perinatal setting.

## FINANCIAL AND MATERIAL SUPPORT

No financial support was received.
